# Non-linear properties of the Achilles tendon determine ankle impedance over a broad range of activations in humans

**DOI:** 10.1242/jeb.244863

**Published:** 2023-07-27

**Authors:** Kristen L. Jakubowski, Daniel Ludvig, Eric J. Perreault, Sabrina S. M. Lee

**Affiliations:** ^1^ Department of Biomedical Engineering, Northwestern University, Evanston, IL 60208, USA; ^2^ Department of Physical Therapy and Human Movement Sciences, Northwestern University, Chicago, IL 60611, USA; ^3^ Shirley Ryan AbilityLab, Chicago, IL 60611, USA; ^4^ Wallace H. Coulter Department of Biomedical Engineering, Emory University and Georgia Tech, Atlanta, GA 30322, USA; ^5^ Department of Physical Medicine and Rehabilitation, Northwestern University, Chicago, IL 60611, USA; ^6^ Department of Biomedical Physiology and Kinesiology, Simon Fraser University, Burnaby, BC, Canada, V5A 1S6

**Keywords:** Muscle, Musculotendon mechanics, System identification, Stiffness, Ultrasound imaging

## Abstract

Regulating ankle mechanics is essential for controlled interactions with the environment and rejecting unexpected disturbances. Ankle mechanics can be quantified by impedance, the dynamic relationship between an imposed displacement and the torque generated in response. Ankle impedance in the sagittal plane depends strongly on the triceps surae and Achilles tendon, but their relative contributions remain unknown. It is commonly assumed that ankle impedance is controlled by changing muscle activation and, thereby, muscle impedance, but this ignores that tendon impedance also changes with activation-induced loading. Thus, we sought to determine the relative contributions from the triceps surae and Achilles tendon during conditions relevant to postural control. We used a novel technique that combines B-mode ultrasound imaging with joint-level perturbations to quantify ankle, muscle and tendon impedance simultaneously across activation levels from 0% to 30% of maximum voluntary contraction. We found that muscle and tendon stiffness, the static component of impedance, increased with voluntary plantarflexion contractions, but that muscle stiffness exceeded tendon stiffness at very low loads (21±7 N). Above these loads, corresponding to 1.3% of maximal strength for an average participant in our study, ankle stiffness was determined predominately by Achilles tendon stiffness. At approximately 20% MVC for an average participant, ankle stiffness was 4 times more sensitive to changes in tendon stiffness than to changes in muscle stiffness. We provide the first empirical evidence demonstrating that the nervous system, through changes in muscle activations, leverages the non-linear properties of the Achilles tendon to increase ankle stiffness during postural conditions.

## Teaser

**Summary:** During postural conditions, the stiffness of the ankle in humans is determined primarily by the stiffness of the Achilles tendon, not that of the triceps surae muscles.

## INTRODUCTION

The ability to adapt the mechanical properties of the ankle is essential for seamlessly transitioning across different terrains when walking and for maintaining postural stability when unexpectedly perturbed (Finley et al., 2012; Whitmore et al., 2019). The triceps surae muscles and the Achilles tendon are the primary determinants of ankle mechanics in the sagittal plane, but their relative contributions remain largely unknown. It is commonly assumed that changes in ankle mechanics during active contractions are largely determined by the activation-dependent properties of muscle (Cook and McDonagh, 1996; Sartori et al., 2015; Weiss et al., 1988), but there have been limited *in vivo* measurements validating this presumption. Determining how the triceps surae and Achilles tendon mechanics contribute to ankle mechanics across a broad range of physiological conditions would provide fundamental insight into the mechanisms underlying humans' ability to navigate their physical world. Such knowledge could also aid in developing targeted interventions when musculotendon mechanics are altered due to neuromuscular pathologies, or in developing biomimetic assistive devices (Sartori et al., 2015; Sartori and Sawicki, 2021). As such, we sought to determine the relative contribution from the triceps surae and Achilles tendon to the mechanics of the ankle.

The presumed primary role of muscle in determining the mechanical properties of the ankle is based on two assumptions. The first is that muscle impedance is substantially lower than tendon impedance for most physiological conditions. The second assumption is that tendon impedance during active conditions is constant across loads and that changes in joint impedance must therefore be due to changes in muscle impedance. Impedance – a quantitative measure of mechanics – describes the dynamic relationship between an imposed displacement and the evoked forces or torques (Kearney and Hunter, 1990).

Owing to the serial connection between the muscle and tendon, ankle impedance will be determined mainly by which of these components has the lowest impedance. The Achilles tendon is long and compliant (Farris and Sawicki, 2012; Fukunaga et al., 2001), and its impedance relative to that of the triceps surae is unknown. Therefore, muscle impedance may not be substantially lower than tendon impedance during physiologically relevant conditions.

Nearly all experimental studies quantifying muscle and tendon mechanics have focused on stiffness, the static component of impedance. It is well known from *in vivo* experiments that muscle stiffness changes with activation-dependent changes in muscle force (Cui et al., 2008; Rack and Westbury, 1974). Several studies have measured tendon stiffness *in vivo* as the slope of the tendon force–length curve, where force is estimated based on the measured ankle torque, and B-mode ultrasound is used to image muscle–tendon junction (MTJ) displacement, which serves as a proxy for changes in tendon length (de Oliveira et al., 2016; Foure et al., 2012; Hauraix et al., 2015; Lichtwark and Wilson, 2005; Theis et al., 2012). These measurements have been made during passive conditions (de Oliveira et al., 2016; Theis et al., 2012), and active contractions (Foure et al., 2012; Hauraix et al., 2015; Lichtwark and Wilson, 2005). However, measurements during active conditions often implicitly assume that tendon stiffness remains constant across loads as the slope of the tendon force–elongation curve is commonly measured over a wide range of activations (e.g. 50–90%) (Foure et al., 2012; Kubo et al., 2002). While it is reasonable to assume tendon stiffness is constant at high loads (above approximately 30% of maximum force) (Proske and Morgan, 1987; Zajac, 1989), tendons have a non-linear stress–strain relationship that results in load-dependent stiffness properties in the lower load regime within the ‘toe region’ (Abrahams, 1967; Lewis and Shaw, 1997; Proske and Morgan, 1987; Zajac, 1989). To our knowledge, only one study has used this ultrasound method to quantify tendon stiffness at lower levels of muscle activation (Maganaris and Paul, 2002). Ultimately, accounting for the non-linear properties of the tendon and making measurements across the full range of loads could impact the relative contributions from the muscle and tendon to the stiffness of the joint.

There is conflicting experimental evidence on how triceps surae and Achilles tendon stiffness vary with respect to each other and, therefore, on their relative contributions to ankle stiffness. This stems from the fact that few studies have examined muscle and tendon stiffness over a wide range of loads relevant to common functional tasks. Previously, during passive stretching, it was observed that the Achilles tendon takes up the majority of the length change of the muscle–tendon unit (MTU), suggesting that it is less stiff than the muscle (Herbert et al., 2002). During active contractions above 30% of the maximum voluntary contraction (MVC), it was observed that tendon stiffness is greater than muscle stiffness (Hauraix et al., 2015). In contrast, others have observed that the Achilles tendon is more compliant than the triceps surae during standing (Loram et al., 2007), which typically occurs around 15% MVC (Nagai et al., 2011). The conflicting results during active contractions may be due partly to differences in the tested range of muscle activations and differences in the experimental paradigms. To our knowledge, no one has bridged the gap between these estimates during passive and active conditions and quantified the relative contribution from the muscle and tendon across a range of activations (and forces) that are relevant to many functional tasks.

The objective of this study was to determine how the triceps surae and Achilles tendon contribute to the impedance of the ankle during conditions relevant to postural control. We used an innovative technique that combines joint-level perturbations with B-mode ultrasound to quantify ankle, muscle and tendon impedance (Jakubowski et al., 2022). Given the limited and conflicting experimental data reported in the literature, we tested the null hypothesis that the muscle and tendon contribute equally to ankle impedance to determine which structure was most dominant over contraction levels ranging from 0% to 30% MVC. Our results help determine the mechanisms contributing to the regulation of human ankle impedance, as needed for seamless interactions with the environment. As a secondary objective, we quantified the frequency ranges over which muscles and tendons behave elastically. Though there are conditions for which muscles and tendons exhibit spring-like behavior, both structures are viscoelastic (Moss and Halpern, 1977; Peltonen et al., 2013). Until recently (Jakubowski et al., 2022), it has not been possible to quantify muscle and tendon impedance *in vivo* in humans. As such, it is unknown under which conditions it is reasonable to assume that muscles and tendons behave as simple springs with only a stiffness component and when they exhibit more complex mechanical properties. We, therefore, quantified these regimes in this study.

## MATERIALS AND METHODS

### Participants

Seventeen healthy young adults (mean±s.d. age 27±3 years, height 1.7±0.1 m, body mass 73±15 kg; 8 males and 9 females) participated in this experiment. All participants were right-leg dominant and had no history of neuromuscular or musculoskeletal injuries to their right leg. All participants provided written informed consent prior to participation. The Northwestern University Institutional Review Board approved the study, and all methods were carried out according to the approved protocols (STU00009204 and STU00213839).

### Experimental setup

**Fig. 1.**
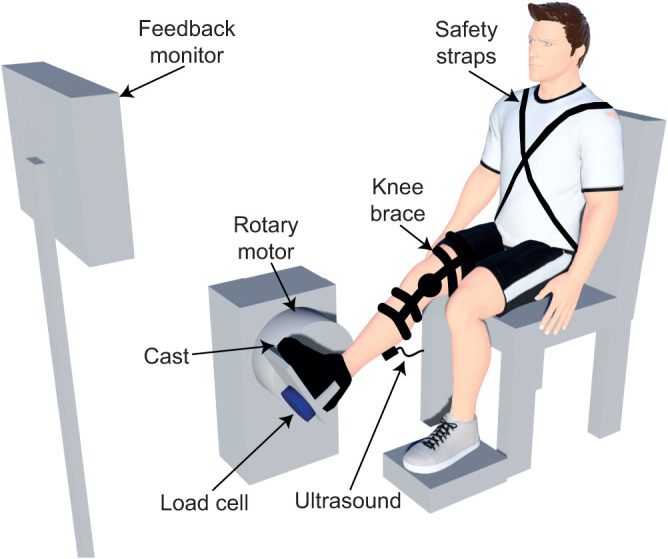
**Schematic diagram of the experimental setup.** A custom-made cast secured the participant's foot to the rotary motor. The rotary motor rigidly controlled the ankle joint angle, while the load cell measured the resultant ankle torque. We used B-mode ultrasound to image the muscle–tendon junction (MTJ) of the medial gastrocnemius. The knee brace secured the knee in a stable position, preventing unwanted knee flexion or extension. The feedback monitor provided real-time feedback on the magnitude of the plantarflexion torque and the tibialis anterior muscle activity.

Participants were seated in an adjustable chair (Biodex Medical Systems, Inc., Shirley, NY, USA), with their trunk and torso stabilized with safety straps (Fig. 1). The participant's right leg was extended in front of them with the knee flexed at 15 deg. A knee brace (Innovator DLX, Ossur, Reykjavik, Iceland) stabilized the knee in this position. The participant's right foot was attached rigidly to an electric rotary motor (BSM90N-3150AF, Baldor, Fort Smith, AR, USA) via a custom-made fiberglass cast at an ankle angle of 90 deg. The cast encased the entire foot, extending distally from the medial and lateral malleoli to the toes, thus preserving the full range of motion of the ankle but preventing any movement of the foot or toes. The axis of rotation of the motor was aligned with the ankle center of rotation in the sagittal plane, restricting all movement to the plantarflexion/dorsiflexion direction. Electrical and mechanical safety stops limited the rotation of the motor within the participant's range of motion. A 24-bit quadrature encoder integrated with the motor measured ankle angle (24-bit, PCI-QUAD04, Measurement Computing, Norton, MA, USA), while a 6-degree-of-freedom load cell (45E15A4, JR3, Woodland, CA, USA) measured all ankle forces and torques. Throughout the experiment, the motor was controlled in real time via xPC target (MATLAB, MathWorks, Natick, MA, USA).

Single differential bipolar surface electrodes (Bagnoli, Delsys Inc., Boston, MA, USA) measured muscle activity from the medial and lateral gastrocnemius and soleus (ankle plantarflexors) and the tibialis anterior (ankle dorsiflexor). Standard skin preparation methods were performed prior to electrode placement (Tankisi et al., 2020), and electrodes were placed on the belly of the muscle. Electromyography (EMG) signals were amplified to maximize the signal resolution in each channel. EMG data were collected for visual feedback provided to the participants. All analog data were passed through an antialiasing filter (500 Hz using a 5-pole Bessel filter) and sampled at 2.5 kHz (PCI-DAS1602/16, Measurement Computing). EMG data were collected and used to provide visual feedback to the participant.

A B-mode ultrasound system using a linear transducer (LV7.5/60/128Z-2, LS128, CExt, Telemed, Vilnius, Lithuania) recorded images of the medial gastrocnemius MTJ. A custom-made probe holder and elastic adhesive wrap (Coban™, 3M, St Paul, MN, USA) secured the probe to the leg. We positioned the ultrasound probe to center the MTJ on the image. At the start of ultrasound data collection, a trigger signal was used to synchronize the ultrasound data collection with all other measurements. Ultrasound images were acquired with a mean frame rate of 124 Hz. All ultrasound data were saved for processing offline.

### Protocol

At the start of each experiment, we collected three 10 s isometric MVC trials in both the plantarflexion and dorsiflexion directions. These data were used to scale the visual feedback provided to the participants.

Our primary objective was to determine how muscle, tendon and ankle impedance vary across various levels of plantarflexion torque. This was accomplished by instructing participants to produce different levels of isometric plantarflexion torque while the rotary motor applied small rotational perturbations in the sagittal plane. We used pseudo-random binary sequence (PRBS) perturbations with an amplitude of 0.175 radians, a maximum velocity of 1.75 radians per second, and a switching time of 153 ms. We tested seven isometric plantarflexion torque levels from 0% to 30% MVC in 5% increments. Participants were provided with real-time visual feedback of their normalized plantarflexion torque. Tibialis anterior EMG was also provided to prevent co-contraction. Rectified EMG and torque signals were low-pass filtered at 1 Hz to remove high-frequency components from the applied perturbations (2nd order Butterworth). Participants completed three trials at each level of plantarflexion torque in a randomized fashion. Each trial lasted 65 s. Rest breaks were provided as needed between trials to prevent fatigue.

The measured ankle torque included the gravitational and inertial contributions from the apparatus connecting the foot to the motor. A single trial was collected with only the cast attached to the rotary motor enabling us to remove these contributions from the net torque measured in each trial.

### Data processing and analysis

All data were processed and analyzed using custom-written software in MATLAB. The same individual manually digitized the MTJ within each frame of the ultrasound videos (Jakubowski et al., 2022). All ultrasound metrics were resampled using linear interpolation to match the sampling rate of the other experimental signals (2.5 kHz).

**Fig. 2.**
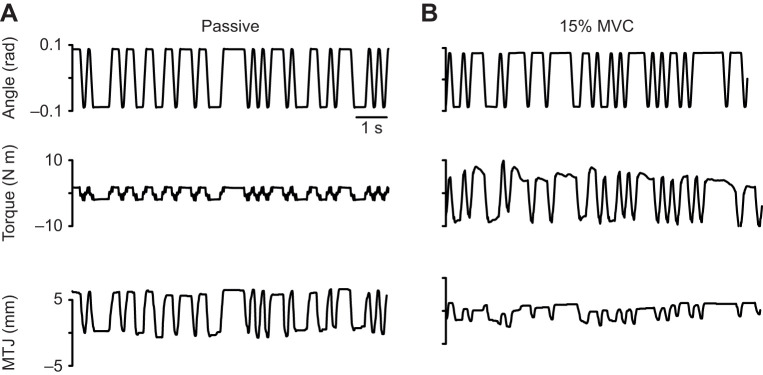
**Representative data used to estimate ankle, muscle and tendon impedance.** Representative data from a passive trial (A) and a trial when the participant was instructed to maintain 15% of their maximum voluntary torque (MVC) (B). The rotary motor rigidly controlled the position of the participant's ankle (angle) at all times. We measured the resultant ankle torque and MTJ displacement from the medial gastrocnemius resulting from the applied random perturbations. Torque and MTJ displacement have been detrended.

We computed ankle, muscle and tendon impedance as described previously (Jakubowski et al., 2022). Briefly, we used non-parametric system identification to estimate ankle, muscle and tendon impedance from the experimental measures of ankle angle, ankle torque and displacement of the MTJ (Fig. 2). We quantified ankle impedance as the relationship between the imposed ankle rotations and the resultant ankle torque (Kearney and Hunter, 1990). Measurement of the MTJ motion allowed us to estimate muscle and tendon impedance under the assumption that the muscle and tendon are connected in series (Hill, 1938), and that the displacement of the MTU is determined by the angular rotation of the ankle multiplied by the Achilles tendon moment arm. Our method also assumes that the proximal end of the muscle is fixed, and any movement of the MTJ is a measure of the change in muscle length. Thus, we can estimate muscle and tendon impedance from estimates of ankle impedance and the translation ratio – the relationship between MTJ displacement and the angular rotations of the ankle. Specifically, to characterize ankle, muscle and tendon impedance, we estimated ankle impedance and the translation ratio, and used these quantities to compute muscle and tendon impedance (Jakubowski et al., 2022). We previously demonstrated that the magnitude of the frequency response functions was nearly constant from 1 to 3 Hz and had a high coherence (Jakubowski et al., 2022). This indicates that stiffness was the dominant contributor to impedance over this frequency range, and our data had a high signal-to-noise ratio (Jakubowski et al., 2022). Additionally, this frequency range (1–3 Hz) is relevant to common tasks, including locomotion (Angeloni et al., 1994). As such, we computed the stiffness component of ankle, muscle and tendon impedance by averaging the magnitude of the respective frequency response functions from 1 to 3 Hz. Our primary analysis will focus on the stiffness component of impedance due to its relevance in the control of posture and movement at the ankle (Ludvig et al., 2022).

A single approximation of the Achilles tendon moment arm (51.4 mm) was used for all analyses. This was estimated as the mean across participants from Clarke et al. (2015) with an ankle angle of 90 deg. It has been demonstrated that the Achilles tendon moment arm does not scale with anthropometric data (Clarke et al., 2015; Sheehan, 2012). Additionally, system identification is a quasi-linear approximation about a single operating point, which, in our study, was 90 deg. Therefore, we approximated the moment arm as a single value.

Ankle and tendon stiffness – the low-frequency component of impedance – varied non-linearly with plantarflexion torque (or musculotendon force). Therefore, the ankle and tendon stiffness experimental data were fitted with non-linear models to synthesize our results. The model used to characterize torque-dependent changes in ankle stiffness was:
(1)

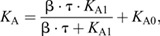
in which *K*_A_ represents the modeled ankle stiffness, torque (τ) was the input to the model, and β, *K*_A1_ and *K*_A0_ are the optimized parameters. A similar model has been used to characterize load-dependent changes in the stiffness of a MTU (Morgan, 1977).

Tendon stiffness was modeled by an exponential function:
(2)

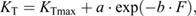
in which *K*_T_ represents the modeled tendon stiffness, musculotendon force (*F*) was the input to the model, and *K*_Tmax_, *a* and *b* are the optimized parameters. This model was chosen because exponential models have been used previously to characterize the non-linear toe region of the tendon stress–strain curve (Lichtwark and Wilson, 2008). We computed musculotendon force by dividing the measured ankle torque by the Achilles tendon moment arm. We note that this non-linear model of tendon properties was not incorporated into Eqn 1. This simplification was made for two reasons. The first is the prior use of Eqn 1 in the literature (Cui et al., 2008; Morgan, 1977) and its good fit to our data. The second is that we were unsuccessful in fitting a model of ankle stiffness that included the non-linear mechanics of the tendon; high parameter covariance led to poor convergence.

### Sensitivity analyses

We evaluated the sensitivity of ankle stiffness to changes in muscle and tendon stiffness at different levels of force. We first consider that ankle stiffness (*K*_A_) is determined by the serial connection of the muscle and tendon and can be described as a function of these stiffnesses (Jakubowski et al., 2022), such that:
(3)

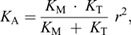
where *r* represents the Achilles tendon moment arm in the sagittal plane, *K*_M_ represents muscle stiffness and *K*_T_ represents tendon stiffness. Note that this equation has the same form as Eqn 1, except that the constant term has been omitted as it does not affect the sensitivity analyses. This relationship was used to derive the sensitivity of ankle stiffness to muscle and tendon stiffness using Eqn 4, where *S_x_* is the relative sensitivity to a given parameter *x* (either muscle or tendon stiffness):
(4)

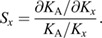
The average values of muscle and tendon stiffness estimated from our experiment were used to compute numerical values for the sensitivity of ankle stiffness.

### Statistical analysis

We sought to determine how the triceps surae and Achilles tendon contribute to the impedance of the ankle over a range of activation levels. Non-linear mixed-effects models were used to characterize the ankle stiffness–torque relationship and the tendon stiffness–force relationship (Eqns 1 and 2). A linear mixed-effects model was used to describe the muscle stiffness–musculotendon force relationship. For all models, subject was treated as a random factor, and plantarflexion torque or musculotendon force was a continuous factor. A restricted maximum likelihood method was used to estimate all models (Luke, 2017). The model fit for ankle, muscle and tendon stiffness was assessed by quantifying the coefficient of determination (*R*^2^) for each participant from the respective mixed-effects model. We tested the null hypothesis that the muscle and tendon contribute equally to ankle stiffness. We used a bootstrapping procedure to determine the range of musculotendon forces when muscle and tendon stiffness were not significantly different from each other to a level of *P*>0.05. The bootstrapping involved randomly resampling the data from each participant with replacement to create a new dataset for the entire pool of participants. This process was repeated 200 times. Each synthesized dataset was analyzed as described above to create a distribution of estimates for which muscle and tendon stiffnesses were the same. Our null hypothesis – that muscle and tendon stiffness contribute equally to the stiffness of the ankle – was accepted within the 95% confidence intervals of this distribution and rejected elsewhere. All metrics reported are means±95% confidence intervals unless otherwise noted.

## RESULTS

### Muscle stiffness exceeded tendon stiffness at low loads

**Fig. 3.**
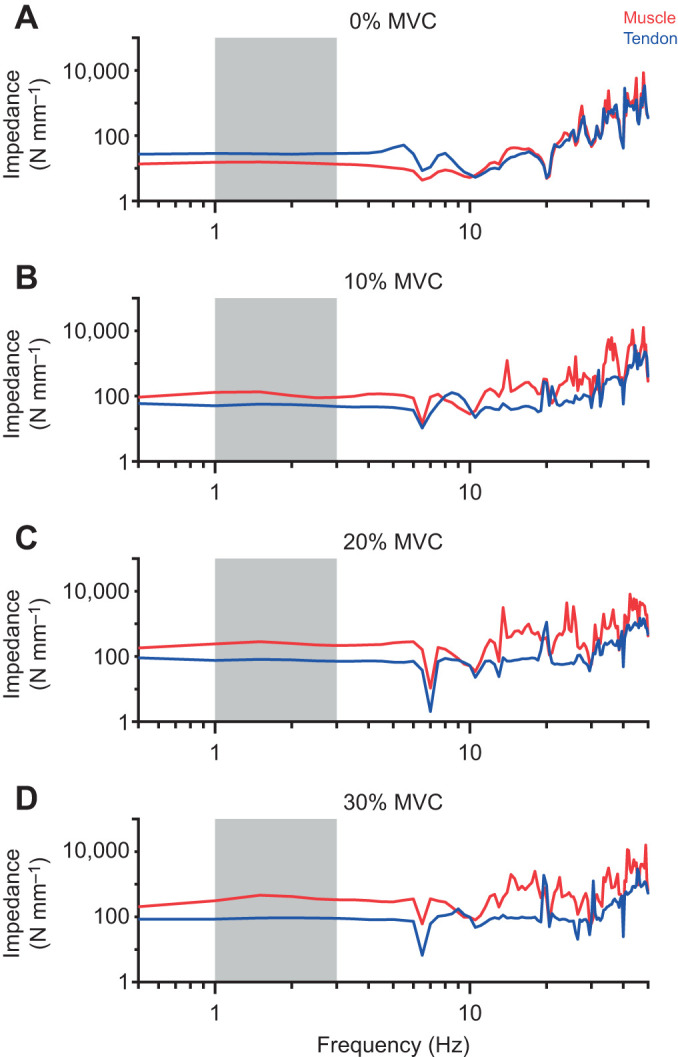
**Stiffness is the dominant contributor to muscle and tendon impedance at low frequencies.** Muscle (red) and tendon (blue) impedance frequency response functions from a representative participant at (A) 0% MVC, (B) 10% MVC, (C) 20% MVC and (D) 30% MVC. The magnitudes of the frequency response functions were nearly constant from 1 to 6.5 Hz, indicating that stiffness is the primary contributor to impedance. Values between 1 and 3 Hz were used to compute stiffness (shaded region).

At all levels of activation, the magnitudes of the frequency response functions for muscle and tendon impedance were nearly constant from 1 to 6.5 Hz (Fig. 3), indicating that stiffness is the primary contributor to impedance at these frequencies. Therefore, it is reasonable to assume that muscle and tendon behave as simple springs during the constant torque conditions tested. Moreover, the frequency range in which muscle and tendon exhibit spring-like behavior was not altered by changes in load.

**Fig. 4.**
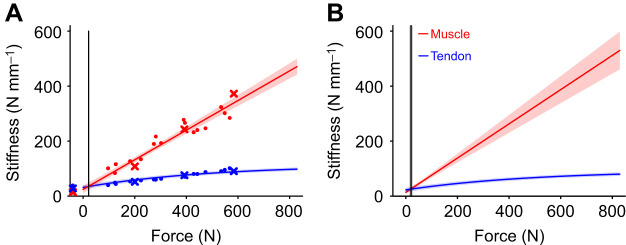
**Muscle stiffness exceeded tendon stiffness past the lowest levels of force.** (A) Muscle stiffness (red) and tendon stiffness (blue) for an individual partcipant, illustrating that muscle stiffness exceeded tendon stiffness at low levels of musculotendon force (21 N, vertical black line). Each point represents an individual trial. The crosses correspond to the trials illustrated in Fig. 3. (B) This trend was preserved across all participants (*n*=17). Muscle stiffness exceeded tendon stiffness at 21±7 N. The tendon stiffness experimental data were modeled using Eqn 2, while the muscle stiffness experimental data were modeled linearly. Mixed-effects models were used for muscle and tendon stiffness to account for random variability between participants. For both plots, the solid line indicates the estimated muscle and tendon stiffness from the respective mixed-effects models, with the shaded region being the 95% confidence interval (CI). We evaluated the range of musculotendon forces when muscle and tendon stiffness were not significantly different from each other to a level of *P*>0.05 using a bootstrapping procedure. The solid black line is the mean musculotendon force where muscle and tendon stiffness were equivalent within the set level of statistical significance, with shading indicating the 95% CI across the bootstrapped samples.

Muscle and tendon stiffness increased with increases in musculotendon force (Fig. 4). Fig. 4A displays the experimental measures and model fits from an individual participant. The muscle and tendon stiffness models fit the data well for the representative participant (muscle: *R*^2^=0.94; tendon: *R*^2^=0.91; Fig. 4A), and across the entire group (muscle: *R*^2^=0.94±0.01; tendon: *R*^2^=0.94±0.01; Fig. 4B).

We found that muscle stiffness increased at a greater rate with increases in force than did tendon stiffness. Data for a representative participant shown in Fig. 4A illustrate that muscle stiffness was greater than tendon stiffness at 21 N (vertical line). This trend was consistent across all participants. We observed that muscle stiffness exceeded tendon stiffness at a musculotendon force of 21±7 N (Fig. 4B); this occurred at a very low contraction level, corresponding to 1.5±0.2% of the maximum voluntary torque across all participants. At the highest force tested in this study, ∼830 N, the muscle was approximately 6.6 times stiffer than the tendon.

### Ankle stiffness was most sensitive to changes in tendon stiffness

**Fig. 5.**
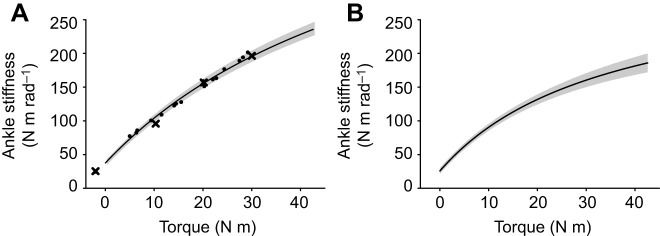
**Ankle stiffness increased with increased plantarflexion torque.** (A) Ankle stiffness estimates for an individual participant, illustrating the increase in stiffness with torque. Each point represents an individual trial. The crosses correspond to the trials illustrated in Fig. 3. This trend was preserved in the group results (*n*=17) (B). The ankle stiffness experimental data were modeled using Eqn 1. A mixed-effects model was used to account for random variability between participants. For both plots, the solid line indicates the estimated stiffness from the respective fitted model, with the shaded region being the 95% CI.

A unique feature of our measurement technique is that we were able to quantify ankle, muscle and tendon stiffness simultaneously, enabling us to quantify the relative contributions from the muscle and tendon to the stiffness of the joint. As others have reported (Hunter and Kearney, 1982; Kearney and Hunter, 1990; Weiss et al., 1988), we found that ankle stiffness increased with voluntary contraction (Fig. 5). This increase was non-linear and described well by Eqn 1 for individual partcipants (Fig. 5A; *R*^2^=0.99), and the full population of tested partcipants (Fig. 5B; *R*^2^=0.98±0.007). Our values of ankle stiffness are consistent with previous reports using a similar experimental protocol (Kearney and Hunter, 1982).

We completed a sensitivity analysis to quantify how changes in muscle and tendon stiffness influence ankle stiffness across the range of tested forces. As expected, ankle stiffness was most sensitive to the tendon for forces above 21 N, where tendon stiffness became lower than muscle stiffness. For forces above 350 N, corresponding to approximately 20% MVC in our population of participants, ankle stiffness was nearly 4 times more sensitive to changes in tendon stiffness than to changes in muscle stiffness. The importance of tendon stiffness for determining ankle stiffness increased at further contraction levels. These results provide additional evidence that the mechanical properties of the human ankle are determined primarily by the non-linear mechanical properties of the Achilles tendon.

## DISCUSSION

**Fig. 6.**
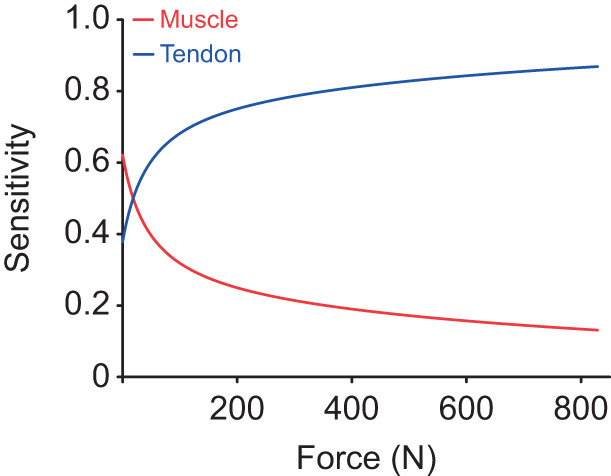
**Sensitivity of ankle stiffness to changes in muscle (red) and tendon (blue) stiffness.** Beyond the lowest levels of force, ankle stiffness was more sensitive to changes in tendon stiffness than to changes in muscle stiffness.

Regulating ankle impedance is critical when adapting to varying environmental conditions and responding to postural disturbances. This study sought to determine how the triceps surae and Achilles tendon contribute to sagittal plane ankle impedance – the dynamic relationship between an imposed displacement and the instantaneous torque evoked in response (Kearney and Hunter, 1990) – over a range of activation levels. We used our novel technique to quantify ankle, muscle and tendon impedance simultaneously (Jakubowski et al., 2022). We found that both muscle and tendon impedance increased with activation, and the stiffness of these structures was the dominant contributor to their impedance below approximately 6.5 Hz. Muscle stiffness, the static component of impedance, exceeded tendon stiffness beyond the lowest forces and levels of activation (∼21 N or ∼1.5±0.2% MVC; Fig. 4). The stiffness of the human ankle during plantarflexion is determined largely by the net stiffness of the serially connected Achilles tendon and triceps surae muscles. Because springs connected in series have a net stiffness that is limited by the most compliant (least stiff) element, our results indicate that the mechanical properties of the Achilles tendon, a passive structure, have a substantial impact on the activation-dependent increases in ankle stiffness at almost all levels of muscle activation, with the ankle being more sensitive to changes in tendon stiffness than to changes in muscle stiffness (Fig. 6). This finding is in contrast to a common assumption that the regulation of ankle stiffness is directly linked to activation-dependent changes in muscle stiffness (Cook and McDonagh, 1996; Sartori et al., 2015; Weiss et al., 1988). Instead, our results demonstrate that the nervous system, through changes in muscle activation, leverages the non-linear properties of the Achilles tendon to increase ankle stiffness across all tested loads. This ability may simplify control compared with the alternative strategy of regulating ankle stiffness by changing the complex mechanical properties of the muscle, which vary with the state of activation, force, length and velocity, among other things.

### Characteristics of triceps surae and Achilles tendon impedance

We found that muscle and tendon have spring-like properties below approximately 6.5 Hz, as indicated by the nearly constant-valued impedance frequency response functions (Fig. 3). This result implies that viscous and inertial properties of the muscle and tendon are small relative to stiffness over this frequency range. This result is consistent with testing in excised tendons, where it has been found that the estimated tendon mechanical properties are invariant with respect to the frequency of the applied stretch up to 11 Hz (Bennett et al., 1986; Schechtman and Bader, 1994). It is also consistent with previous findings within feline muscle, where the muscle behaves elastically in response to small stochastic perturbations over a similar frequency range to that used here (Kirsch et al., 1994). Our measured response in muscle suggests that our measurements remained within its short-range stiffness region (Rack and Westbury, 1974). Muscle short-range stiffness describes the initial response to small, fast displacements prior to reflexive or volitional muscle activation and is critical in the control of posture and limb stability (Kirsch et al., 1994; Perreault et al., 2004). This finding is consistent with our previous results that demonstrated that our muscle stiffness estimates are similar to measurements of muscle short-range stiffness scaled to the triceps surae (Jakubowski et al., 2022). We do note, however, that if the stretch within the muscle or tendon was larger or slower, we would expect to observe more complex viscoelastic behavior. For example, when a larger stretch is applied to a muscle, the response is no longer purely elastic (Rack and Westbury, 1974). Similarly, within tendon, when stretch velocity is slower, the mechanical properties of the tendon decrease (Pearson et al., 2007).

Our estimated values of muscle stiffness were larger than those from the few previous reports that attempted to quantify the stiffness of the human triceps surae muscle *in vivo.* This is likely because of the small size of our perturbations compared with those of earlier studies. All previous estimates of human triceps surae muscle stiffness used perturbations at least twice as large as those we applied (20 deg or larger) (Clark and Franz, 2019; Hauraix et al., 2015). Previously, Hauraix et al. (2015) reported a triceps surae muscle stiffness value of 218 N mm^−1^ at 40% MVC, while Clark and Franz (2019) reported a muscle stiffness of 118 N mm^−1^ at 25% MVC. For comparison, we estimate muscle stiffness to be 261 N mm^−1^ at 25% MVC for an average participant in our study. Muscle stiffness varies based on the size of the applied perturbation (Rack and Westbury, 1974). Therefore, given the difference in perturbation size, it was expected that the previously reported muscle stiffness values would be lower than our results. Given that muscle stiffness scales with force (Cui et al., 2008), it is also possible that differences in muscle stiffness across the studies are related to differences in the load on the muscle. Comparisons at matched forces are not possible across these studies. Our novel *in vivo* estimates of muscle stiffness may be especially pertinent for stability and the response to unexpected postural disturbances, when the short-range stiffness of the muscle has been proposed to be important (De Groote et al., 2017). We also want to highlight that our estimates of muscle stiffness include all structures proximal to the MTJ, including the aponeurosis (Cui et al., 2008; Epstein et al., 2006) and connective tissues (Meyer and Lieber, 2011; Reyna et al., 2020). These contributions can be substantial and are certainly relevant to the mechanical properties of the entire MTU.

The observed increase in tendon stiffness with increases in musculotendon force suggests that the Achilles tendon was within the non-linear toe region of its stress–strain curve during our experiments (Fig. 4). Tendons exhibit a strain-dependent increase in stiffness at low strains (e.g. the toe region of the stress–strain curve) (Zajac, 1989). While Achilles tendon stiffness has been characterized before (Foure et al., 2012; Hauraix et al., 2015; Lichtwark and Wilson, 2005), nearly all previous *in vivo* studies conducted during active conditions have only estimated its stiffness above 30% MVC to satisfy the methodological assumption that tendon stiffness is constant. Our approach is not constrained by this assumption, allowing measurements to be made at lower forces corresponding to activation levels that occur during everyday activities such as standing and walking (Nagai et al., 2011). We do note, however, that while we saw an increase in tendon stiffness across the entire range of activation levels tested, we are within the activation range that is typically considered the toe region (below 30% MVC) (Proske and Morgan, 1987; Zajac, 1989). We speculate that at higher forces (above 30% MVC), the tendon would still be the dominant contributor to ankle stiffness as its stiffness is expected to plateau at higher forces, unlike the stiffness of muscle (Cui et al., 2008; Proske and Morgan, 1987; Zajac, 1989). Future work should investigate the relative contributions of muscle and tendon to ankle mechanics at higher loads to confirm this expectation.

Similar to our main conclusion that the Achilles tendon is the dominant contributor to ankle stiffness, Loram et al. (2007) concluded that the Achilles tendon was less stiff than the muscle. Our results build upon their findings by demonstrating that this result holds across a wide range of loads (Figs 4 and 6). There are also some critical differences between our results and those presented by Loram et al. (2007). While they found that the Achilles tendon was the dominant contributor during small perturbations (∼1 deg), they concluded that during larger perturbations (7 deg), the stiffness of the contractile and series elastic elements were approximately the same. In contrast, we applied 10 deg perturbations throughout our experiment and found that at nearly all levels of muscle activation, muscle stiffness (contractile element) was greater than that of the Achilles tendon (series elastic element) (Fig. 4). The differing results could be related to differences in how muscle and tendon stiffness were estimated. We used direct measures of MTJ displacement in our estimates of muscle and tendon impedance, while Loram et al. (2007) quantified muscle fascicle displacement and relied on a Hill-type muscle model to separate ankle stiffness into the contractile and series elastic components.

### Limitations

Our technique for estimating muscle and tendon stiffness assumes that all plantarflexion torque is transmitted through the Achilles tendon to the triceps surae, omitting contributions from other structures that span the joint (e.g. the joint capsule and other MTUs) (Jakubowski et al., 2022). This assumption is valid during plantarflexion contractions, when the musculotendon force from the triceps surae is significantly greater than contributions from other sources. However, other structures can have a substantial effect relative to Achilles tendon force when the ankle is passively dorsiflexed. To mitigate their contributions, we positioned the ankle in a neutral position where passive torque is minimal (Riener and Edrich, 1999). We may still be overestimating muscle and tendon stiffness during passive conditions, but this limitation will have a negligible impact on our main conclusions when the triceps surae are active. Additionally, assuming that all torque is transmitted through the Achilles tendon ignores the inertial properties of the foot that would contribute to the measured torque. However, we are estimating muscle and tendon stiffness from 1 to 3 Hz, where the magnitude of the frequency response functions were nearly constant (Fig. 3), indicating that the inertia of the foot did not affect our estimates of stiffness or our main conclusions (Jakubowski et al., 2022).

Lastly, our measurement technique assumes that we are estimating the net impedance of the triceps surae and Achilles tendon, despite only making ultrasound measurements at the medial gastrocnemius MTJ. However, we have previously validated this assumption and found that our estimates during active contractions were similar when imaging the medial gastrocnemius, lateral gastrocnemius and soleus MTJs (Jakubowski et al., 2022).

### Functional implications and conclusions

While the data presented were obtained during isometric conditions, our findings may explain an underlying physiological mechanism of previous estimates of ankle impedance during walking. Rouse et al. (2014) observed that ankle stiffness estimated using perturbations of ankle posture during the stance phase of walking was similar to that estimated by the slope of the ankle torque–ankle angle relationship, also known as quasi-stiffness. This was surprising as these two estimation approaches can only yield the same results if the system is purely elastic and passive (Rouse et al., 2012). However, it is well documented that the triceps surae are active during the stance phase of locomotion (Fukunaga et al., 2001; Rouse et al., 2014). One possible explanation for the Rouse et al. (2014) findings is that ankle stiffness was determined primarily by the Achilles tendon – a passive elastic structure – during the stance phase of walking, where it has been shown that muscle fascicle length changes are modest (Farris and Sawicki, 2012; Fukunaga et al., 2001), as in our postural experiment.

We observed that the Achilles tendon is less stiff than the triceps surae at almost all loads, but these results may not apply to other MTUs. For the Achilles tendon, the compliance of the tendon is essential for the storage and return of elastic energy, increasing the economy of locomotion (Biewener and Roberts, 2000; Farris and Sawicki, 2012; Fukunaga et al., 2001; Lichtwark and Wilson, 2007). However, the mechanical properties of the muscle relative to the tendon will depend upon the functional role of each MTU and its corresponding architecture (Lieber and Fridén, 2000). For example, muscles that have a similar fascicle length and tendon slack length have been termed ‘stiff’, while muscles where the fascicles are much shorter than the tendon – such as the triceps surae – have been termed ‘compliant’ (Zajac, 1989). It is almost certain that muscles in the former category will contribute more to the stiffness of the joint that they cross.

Our results from human participants provide a mechanical explanation for a commonly observed phenomenon in both animal and human movement. Many animals have distal muscles connected to long compliant tendons, similar to the human ankle (Alexander, 2002; Alexander and Vernon, 1975; Bennett et al., 1986). Through *in vivo* sonomicrometry, studies in running guinea fowl (Daley and Biewener, 2003) and hopping wallabies (Biewener et al., 1998) have demonstrated that distal tendons take up the majority of the length change of the MTU. A similar phenomenon of large tendon excursions has also been observed in the triceps surae during human locomotion via *in vivo* ultrasound imaging (Farris and Sawicki, 2012; Fukunaga et al., 2001; Ishikawa et al., 2005). All of these results examining musculotendon length changes are consistent with a tendon that is substantially less stiff than the muscle, as we have now been able to measure directly for human participants.

Finally, our results have implications for targeted rehabilitation. Changes in Achilles tendon stiffness that occur as a result of injury (Obst et al., 2018), or healthy aging (Onambele et al., 2006), will impact ankle stiffness. For example, our results suggest that the previously reported age-related decrease in Achilles tendon stiffness will decrease the stiffness of the ankle for a fixed level of contraction (Stenroth et al., 2012). This decrease could impair the control of posture and movement. To improve balance during tasks that require effective ankle stabilization, altering muscle stiffness through strength training might be less effective than increasing tendon stiffness through high-magnitude loading (Bohm et al., 2015; Mersmann et al., 2017). Ultimately, understanding the relative contributions from the muscle and tendon advances our fundamental understanding of how ankle stiffness is varied for an individual's interactions with their physical world, and aids in developing targeted interventions when musculotendon mechanics are altered as a result of neuromuscular pathologies or aging.
